# Assumptions behind scoring source versus item memory: Effects of age, hippocampal lesions and mild memory problems

**DOI:** 10.1016/j.cortex.2017.01.001

**Published:** 2017-06

**Authors:** Elisa Cooper, Andrea Greve, Richard N. Henson

**Affiliations:** MRC Cognition & Brain Sciences Unit, Cambridge, United Kingdom

**Keywords:** Source monitoring, Source memory, Item memory, Ageing, Multinomial processing tree models, MMP, mild memory problems group, HL, Hippocampal Lesion group, MPT, Multinomial Processing Tree, S1, Source 1, S2, Source 2, *Ds*, probability of remembering source, *Di*, probability of remembering item, *Gs*, probability of guessing item's source as S1, *Gi*, probability of guessing an item is old, *Db*, probability of retrieving information about source and item, *Dn*, probability of rejecting an unstudied item, *Dh*, probability of veridical recollection, *Df*, probability of false recollection, *Dm*, probability of missed encoding, 1HT/2HT, one- or two-High-Threshold, LT, low threshold, SDT, Signal-Detection Theory, AIC, Akaike Information Criterion, BIC, Bayesian Information Criterion

## Abstract

Source monitoring paradigms have been used to separate: 1) the probability of recognising an item (Item memory) and 2) the probability of remembering the context in which that item was previously encountered (Source memory), conditional on it being recognised. Multinomial Processing Tree (MPT) models are an effective way to estimate these conditional probabilities. Moreover, MPTs make explicit the assumptions behind different ways to parameterise Item and Source memory. Using data from six independent groups across two different paradigms, we show that one would draw different conclusions about the effects of age, age-related memory problems and hippocampal lesions on Item and Source memory, depending on the use of: 1) standard accuracy calculation vs MPT analysis, and 2) two different MPT models. The MPT results were more consistent than standard accuracy calculations, and furnished additional parameters that can be interpreted in terms of, for example, false recollection or missed encoding. Moreover, a new MPT structure that allowed for separate memory representations (one for item information and one for item-plus-source information; the Source-Item model) fit the data better, and provided a different pattern of significant differences in parameters, than the more conventional MPT structure in which source information is a subset of item information (the Item-Source model). Nonetheless, there is no theory-neutral way of scoring data, and thus proper examination of the assumptions underlying the scoring of source monitoring paradigms is necessary before theoretical conclusions can be drawn.

## 1 Introduction

Evidence from source monitoring paradigms has been influential in shaping theories of human memory (e.g., Johnson et al., 1993, Mitchell and Johnson, 2000, Mitchell and Johnson, 2009, Slotnick and Dodson, 2005, Yonelinas, 1999). These paradigms present participants with an item (e.g., object or word), and ask them to decide whether they studied it previously, and if so, to distinguish in which of two or more sources it was studied (e.g., spatial location or temporal sequence). Comparisons of several populations, such as young versus older (e.g., Cohen and Faulkner, 1989, McIntyre and Craik, 1987, Schacter et al., 1991, Schacter et al., 1994, Spencer and Raz, 1995), or healthy controls versus amnesic patients (e.g., Schacter et al., 1984, Shimamura and Squire, 1987), have often revealed a dissociation, whereby memory for the source differs between groups, even when memory for the item does not. This has been used to support theories that assume separate processes or systems supporting Item and Source memory (Shimamura and Squire, 1987, Yonelinas, 1999), though the precise pattern of dissociations depends on other factors such as the nature of the source (e.g., Cohen and Faulkner, 1989, Dodson et al., 2007, Hashtroudi et al., 1989, Johnson et al., 1993, McIntyre and Craik, 1987, Simons et al., 2004).

In the context of ageing, a related idea is the associative memory hypothesis of Naveh-Benjamin (2000), which proposes that memory problems in older individuals stem from difficulties in associating distinct pieces of information. This theory subsumes source memory, since the age-related source memory deficits that occur under many circumstances are interpreted as the failure to link an item with its source (Bender et al., 2010, Chalfonte and Johnson, 1996, Naveh-Benjamin, 2000, Naveh-Benjamin et al., 2007, Naveh-Benjamin et al., 2004). Nonetheless, under some circumstances, such as when older participants are explicitly instructed to use a linking strategy, age-related impairments in associative memory can be ameliorated (Bastin et al., 2014, Naveh-Benjamin et al., 2007).

In the context of patients with brain disorders, individuals with amnesia following medial temporal lobe damage to structures like the hippocampus can sometimes show item memory deficits, but often show disproportionate deficits in source memory (Schacter et al., 1984, Shimamura and Squire, 1987, Yonelinas et al., 2004). Moreover, individuals with frontal lobe damage consistently show source memory deficits with minimal or no item memory deficits, producing a dissociation often more extreme than amnesic patients without frontal lobe lesions (Duarte et al., 2010, Janowsky et al., 1989, Schacter, 1987).

Despite these general patterns, there are, as alluded to above, other important factors that affect the relative size of the deficits in source versus item memory performance (e.g., deficits in source memory only, source memory impaired more than item memory, or both impaired equally), such as type of encoding or retrieval strategy, or the type of stimulus (for review, see Old and Naveh-Benjamin, 2008, Spencer and Raz, 1995). However, a further factor that affects results, but which is often over-looked, is the method of scoring source versus item memory.

### 1.1 Scoring source memory

A typical source monitoring paradigm involves categorising test items into one of three categories: unstudied (New), studied in Source 1 (S1) and studied in Source 2 (S2). Item memory is often estimated by the proportion of studied items called S1 or S2 (Item Hits), perhaps adjusted for guessing by subtracting the proportion of unstudied items called S1 or S2 (Item False Alarms). Source memory is then typically measured by estimating the probability of categorising the source correctly, given that a studied item was recognised (not called New), i.e., the conditional probability of a Source Hit given an Item Hit (Chalfonte and Johnson, 1996, McIntyre and Craik, 1987, Murnane and Bayen, 1996, Siedlecki et al., 2005, Tree and Perfect, 2004). However, as pointed out by several authors (e.g., Batchelder & Riefer, 1990), these conditional estimates of source memory are still influenced by overall recognition performance and by guessing rates. Moreover, markedly different numbers of trials (e.g., Item Hits) per participant can impair estimation of the average conditional probability across participants (Cox & Snell, 1989). Most importantly, however, the assumptions underlying this standard scoring method are rarely made explicit. For example, Item memory and Source memory could be ordered along a single dimension of memory quality, capturing unidimensional theories of memory (see below). One way to formalise assumptions behind the scoring of source monitoring paradigms is to use Multinomial Processing Trees (MPTs) (Batchelder & Riefer, 1990).

There is a long pedigree of research using MPTs, which can be applied to many psychological domains (see Erdfelder et al., 2009, for a review). For example, the one- or two-High-Threshold (1HT/2HT) models of yes/no recognition memory (which can be seen as a special case of source monitoring with only one source), as reviewed by Snodgrass and Corwin (1988), correspond to different MPTs, and these threshold models have since been extended to the more general case of source monitoring (Bayen, Murmane, & Erdfelder, 1996). Similarly, the “process dissociation procedure” developed by Jacoby (1991) is naturally expressed in terms of a MPT, and has been extended by Erdfelder et al. (2009). More generally, there has been much debate about whether performance on such memory tests is best modelled by continuous distributions of memory strength, as assumed by Signal-Detection Theory (SDT), rather than the discrete memory states assumed by MPTs (Klauer & Kellen, 2010). While continuous levels of item memory and even source memory seem more plausible *a priori*, discrete state models have been shown to offer superior fits over continuous (e.g., SDT) models, when using a Minimum Description Length (MDL) index of fit that takes into account differences in the functional forms of the models (in terms of their flexibility; Kellen, Klauer, & Bröder, 2013). In this statistical sense, discrete state models like MPTs may be preferable for comparing estimates across groups, particularly for binary item and source judgements, as is the case in typical source monitoring tasks.

Despite this long pedigree of work on MPT models, less consideration has been given to the theoretical assumptions behind the various possible models (“tree structures”) that can be fit to data from source monitoring paradigms. Theoretically, decisions in a source monitoring task could depend on a single mental representation, with performance determined by the “quality” of that representation for each test item. This “Item-Source” model, in which source memory is a subset of item memory, was the model assumed by the initial papers that introduced MPT of source monitoring (Batchelder and Riefer, 1990, Riefer and Batchelder, 1988). However, little theoretical justification was given for this specific model, and subsequent papers have simply stuck with this model, without considering other alternative theories from the memory literature.

A common alternative to unidimensional memory models are two-dimensional accounts, such as the independent dual-process model of Yonelinas (1999), which assume that source memory depends on a recollection process, whereas item memory can be supported either by recollection or by an independent familiarity process (see DeCarlo, 2007, Hautus et al., 2008, Slotnick et al., 2000, for other two-dimensional accounts of source memory tasks). Recent neurocomputational models of memory propose different neural substrates underlying each type of memory representation, such that the perirhinal cortex uses representations that support familiarity/item memory (pattern matching), while the hippocampus stores representations that support recollection/source memory (pattern completion) (Norman & O'Reilly, 2003). Other neuroanatomical theories propose alternative anatomical foundations, such as medial temporal lobe structures (including both the perirhinal cortex and the hippocampus) supporting item memory, with source memory requiring additional involvement of prefrontal cortex (Schacter, 1987, Shimamura and Squire, 1987). Indeed, the relative sensitivity of prefrontal cortex to ageing has been used to explain why source memory tends to show greater decrements with age than does item memory (Glisky et al., 1995, Glisky et al., 2001, Levine et al., 2002, West, 1996).

**Fig. 1:**
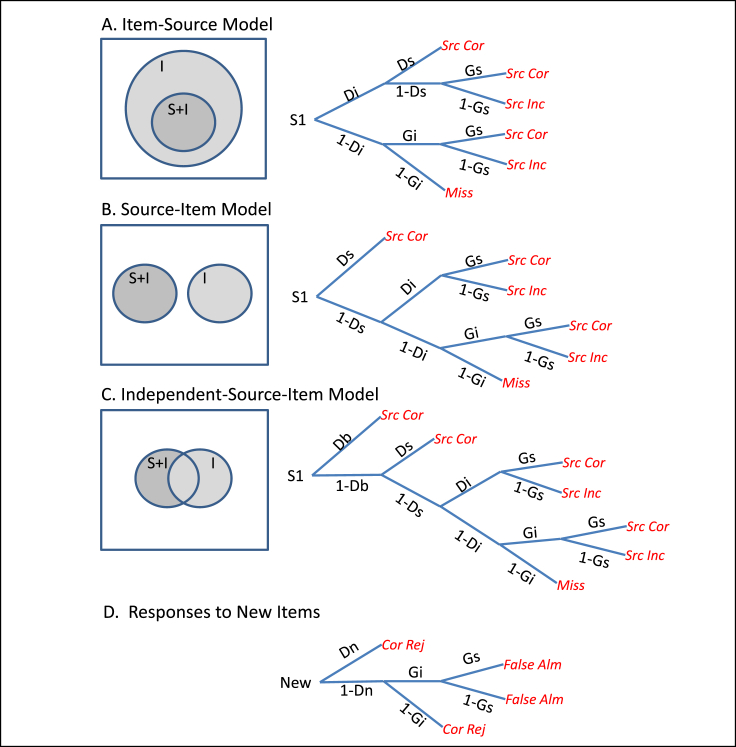
Venn diagrams (left) and Multinomial Processing Trees (MPTs, right) for different memory models. In Venn diagrams, “I” refers to memory system/process underlying Item memory, and “S + I” refers to memory system/process underlying both Source and Item memory. In MPTs, leftmost root of tree denotes trial type and rightmost ends of branches denote various response outcomes. MPTs in Panels A–C distinguish three models in their characterisation of responses to items studied in Source 1 (S1); MPT in Panel D captures responses to unstudied (new) items, and is shared across the three models. The Item-Source Model (A) assumes source memory is a subset of item memory, perhaps subserved by a single memory system/process. The Source-Item Model (B) assumes two memory systems/processes contribute to memory (in an exclusive fashion). The Independent-Source-Item Model (C) assumes two memory systems/processes contribute independently to memory. MPT parameters are: *Ds* = probability of remembering source, *Di* = probability of remembering item, *Gs* = probability of guessing item's source as S1, *Gi* = probability of guessing an item was studied, *Db* = probability of retrieving information about source and item, *Dn* = probability of concluding that an unstudied item is new. Response outcomes are labelled by: Src = Source, Cor = Correct, Inc = Incorrect, Alm = Alarm, Rej = Rejection.

In addition to the number of memory representations, there is the question of how retrieval of one or more of them influences the decisions in a source monitoring paradigm. One possibility is that item information is retrieved first, after which source information may emerge, consistent with the conventional Item-Source MPT model. For example, in an attractor neural network model, a global match between a test cue and a studied item might initially produce a sense of familiarity that can be used to make an item memory decision, and if that match is sufficient, the network might subsequently settle into an attractor that corresponds to a more complete re-instantiation of the study trial (pattern completion), which enables a correct source memory decision (Greve, Donaldson, & Van Rossum, 2010). This corresponds to the MPT model shown in Fig. 1A, where source memory is a subset of item memory. In MPTs, the trial type (in this example, for an item studied in source S1) is the root of the tree, while the various possible response outcomes are the ends of the branches. The parameters (such as *Di*, *Ds*, *Gi*, *Gs* in Fig. 1) correspond to probabilities of certain steps through the tree (from left to right). In this example, *Di* relates to item memory, *Ds* relates to source memory, *Gi* refers to guessing the item was studied and *Gs* refers to guessing the item came from Source 1. More precisely, in the Item-Source model in Fig. 1A, the parameter *Di* estimates the probability of item memory (global match), whereas *Ds* estimates the probability of source memory (pattern completion), given that Item memory occurs (i.e., the proportion of the darker region within the lighter region in the Venn diagram of Fig. 1A). Importantly *Di* includes cases both with and without source memory. In terms of the order of Item and Source decisions, the tree structure shown in Fig. 1A corresponds to the most common, if not only, structure considered in previous applications of MPTs to source monitoring data (e.g., Batchelder and Riefer, 1990, Bayen et al., 1996).

Two-dimensional/dual process accounts of memory, however, raise the possibility of an alternative “Source-Item” *model*, where one process (e.g., involving the hippocampus or prefrontal cortex) can retrieve an episodic representation (recollection) of both the item and its source, whereas a second process (e.g., involving the perirhinal cortex) provides a global match signal that can support item memory (familiarity). One way to represent such a model is shown in Fig. 1B. In this model, the parameter *Ds* estimates the probability of source memory (episodic retrieval), and *Di* now estimates the probability of item memory (global match), given that episodic retrieval does not occur. Importantly *Ds* includes both source and item memory.

Yet further MPTs are possible, for example to allow for the probability (*Db*) that both episodic retrieval and global match occur (as in the independent dual-process model of Yonelinas, 1999), as shown in Fig. 1C, but we do not consider them further because they are over-parameterised for our data (i.e., there are insufficient numbers of response categories, preventing us from estimating their parameters efficiently). For completeness, Fig. 1D shows the MPT for unstudied (new) trials, where “*Dn*” corresponds to the probability of deciding that an unstudied item is new (*Dn* corresponds to the low threshold in a 2HT model).

The MPTs in Fig. 1A and B can fit the data from basic two-source tasks equally well, because they are re-parametrisations of each other (as shown formally in Supplementary Material). This is not always the case, however, as we show later with a paradigm providing more response categories and more complex trees. Moreover, even for isomorphic trees, their parameters can have different meanings, which affect the conclusions one would draw when comparing the parameters across conditions or across groups of participants (e.g., young versus older). For example, if ageing impaired the function of prefrontal cortex or hippocampus, but not perirhinal cortex, then the *Ds* parameter in the Source-Item model (Fig. 1B) would be reduced, but the *Di* parameter (reflecting the conditional probability of global match when episodic retrieval fails) would be unaffected. However, if the same data were fit by the Item-Source model (Fig. 1A), both *Ds* and *Di* parameters would be reduced (see Equations 1 + 2 in Supplementary Material), since *Di* includes both item and source memory.

**Fig. 2:**
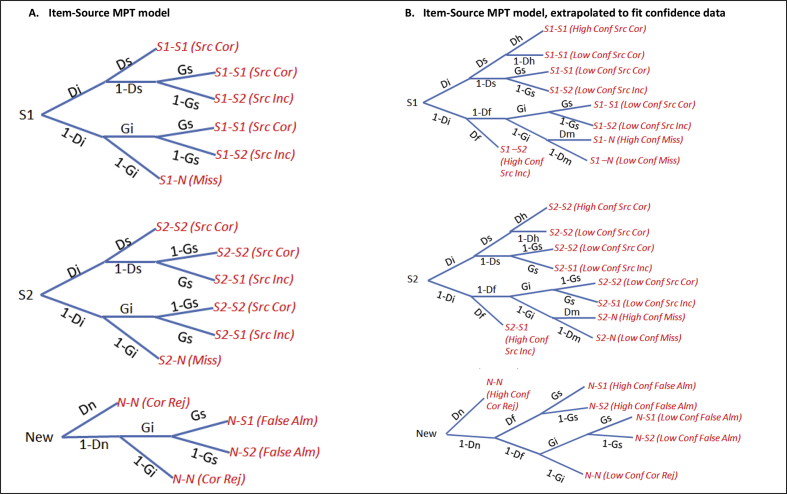
Full Item-Source MPT model from Fig. 1A with separate trees for each source. Panel A shows the Item-Source MPT for Paradigm 1, while Panel B shows the Item-Source MPT for Paradigm 2, which contained additional confidence data. S1 = Source 1, S2 = Source 2, N = New. The response category “S1—S2” means an item studied in Source 1 was judged (incorrectly) as studied in Source 2. *Ds* = probability of remembering source (assumed to be equal for both sources), *Di* = probability of remembering item, *Gs* = probability of guessing item's source as S1, *Gi* = probability of guessing an item was studied, *Dn* = probability of concluding that an unstudied item is new, *Dh* = probability of veridical recollection, *Df* = probability of false recollection, *Dm* = probability of missed encoding, Src = Source, Cor = Correct, Inc = Incorrect, Conf = Confidence, Alm = Alarm, Rej = Rejection.

**Fig. 3:**
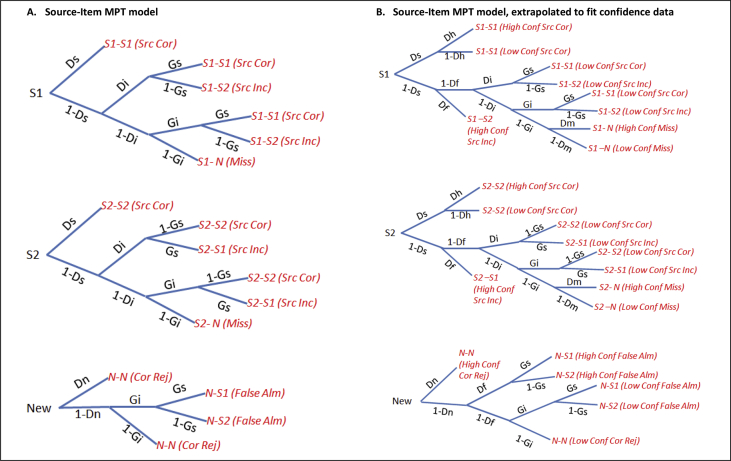
Full Source-Item MPT model from Fig. 1B with separate trees for each source. Panel A shows the Source-Item MPT for Paradigm 1, while Panel B shows the Source-Item MPT for Paradigm 2, which contained additional confidence data. S1 = Source 1, S2 = Source 2, N = New. The response category “S1—S2” means an item studied in Source 1 was judged (incorrectly) as studied in Source 2. *Ds* = probability of remembering source (assumed to be equal for both sources), *Di* = probability of remembering item, *Gs* = probability of guessing item's source as S1, *Gi* = probability of guessing an item was studied, *Dn* = probability of concluding that an unstudied item is new, *Dh* = probability of veridical recollection, *Df* = probability of false recollection, *Dm* = probability of missed encoding, Src = Source, Cor = Correct, Inc = Incorrect, Conf = Confidence, Alm = Alarm, Rej = Rejection.

The full MPT models, with individual trees for each source, are shown for the Item-Source Model in Fig. 2A and for the Source-Item Model in Fig. 3A. These were the MPTs applied to the first paradigm presented here. When a source monitoring paradigm includes confidence ratings, as in the second paradigm presented here, the MPTs can be extended to include additional parameters, as illustrated by the more complex models in Figs. 2B and 3B. These include the parameter *Df* to capture the probability of retrieving an incorrect episode (false recollection), which has been argued to increase with age (Bender et al., 2010, Cohen and Faulkner, 1989, Dodson et al., 2007, Norman and Schacter, 1997) and after prefrontal lesions (confabulations) (Janowsky et al., 1989, Schacter, 1987); the parameter *Dh* to capture the probability of high confidence source hits (e.g., vivid recollection, which might be expected to be higher in Younger people); and the parameter *Dm* to capture the probability of high confidence misses (e.g., owing to missed encoding of a Study trial, which might be expected to be more common if Older people struggle to sustain attention, for example). These extended models are elaborated later when presenting Paradigm 2.

Below, we fit both the Item-Source and Source-Item MPTs to data from three independent groups of participants in each paradigm, in order to demonstrate that different MPT models affect the conclusions one would draw about differences in item and/or source memory across groups. The first paradigm was a continuous monitoring task in which an object was presented on the left or right side of a background scene. This paradigm was run on a group of 18 younger adults, a group of 18 older adults and a group of 3 adults with acquired, focal hippocampal lesions (HL group). The second paradigm was a study-test task in which objects were presented above or below a fixation cross on a blank screen, and included additional confidence ratings for each response. This paradigm was run on a group of 12 younger adults, a group of 12 older adults and a group of 105 older adults with mild memory problems (MMP group).

## 2 Paradigm 1: object-scene

**Fig. 4:**
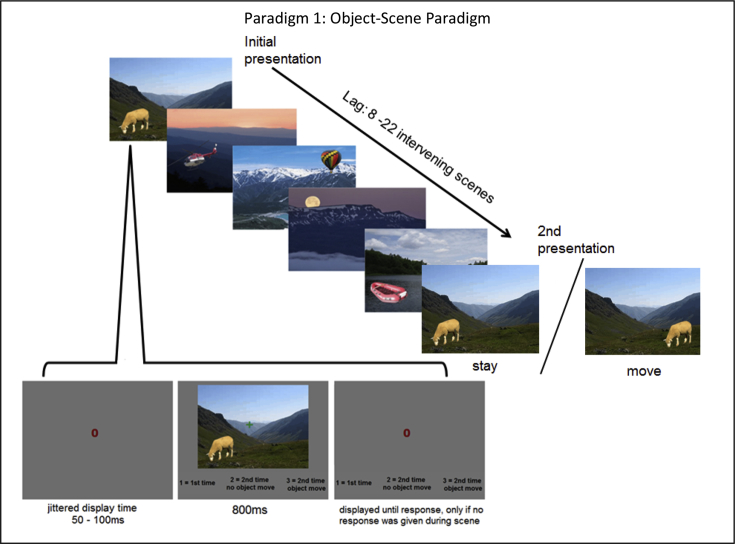
Paradigm 1: Object-Scene Paradigm. This was a continuous source monitoring paradigm, in which an object was presented either to the left or right of the centre of a scene. Each object-scene pair repeated at lags of between 8 and 22 intervening items. For repeated presentations, objects were either on the same left/right side (“stay” condition) or switched side (“move” condition).

Paradigm 1 is shown in Fig. 4. This was a continuous source monitoring paradigm, where foreground objects were presented on background scenes. Each object-scene pair was presented twice, but on one half of the repetitions, the object switched its left-right location on the scene. On each trial, participants made a three-way decision of: “new” (first time object-scene pair seen), “stay” (second time pair seen, with same object location) and “move” (second time pair seen, but object location switched). This paradigm was based on an fMRI study of Howard, Kumaran, Ólafsdóttir, and Spiers (2011) which showed that the hippocampus is particularly active for “move” trials, suggesting that it is important for coding object-scene spatial relationships. To fit the MPT models, S1 was defined as “stay” and S2 was defined “move”, such that *Gs* was the probability of guessing “stay” and 1-*Gs* was the probability of guessing “move”. These assignments are arbitrary, and do not affect the conclusions. Note that the paradigm is not a conventional source monitoring paradigm, because the scenes provide a unique associate for each item (object) (indeed, it could also be considered an “associative memory” paradigm, where the association is between a scene and an object location). However, it can easily be re-parameterised as a source memory paradigm if S1 is assigned to one location on first presentation of a scene (e.g., left, so that S2 corresponds to initial presentations on the right): Then a “stay” decision for a repeated S1 trial with the same object location, or a “move” decision for a repeated S1 trial with the opposite object location, would be considered correct source judgements.

This paradigm was run on a Young and Older group (*N* = 18 each), and *N* = 3 adults with acquired, focal hippocampal lesions (HL group). We expected the Older group to have lower values of *Ds* than the Young Group, given compelling evidence for impaired source retrieval/recollection with age (Hashtroudi et al., 1989, Koen and Yonelinas, 2014, McIntyre and Craik, 1987, Spencer and Raz, 1995), and also possibly lower values of *Di*, though some have claimed that item memory/familiarity can be unaffected by ageing (Koen and Yonelinas, 2014, Naveh-Benjamin, 2000, Schacter et al., 1994). Likewise, given claims that Hippocampus is important for recollection (Yonelinas, 2002), we expected lower *Ds* values in the HL group than Older control group, but not necessarily lower values of *Di*, given claims that extra-Hippocampal regions like Perirhinal cortex can support item memory (Norman and O'Reilly, 2003, Yonelinas, 2002). We did not expect a bias in any group in guessing a particular source (*Gs*), but we were aware that Older people might show a higher *Gi* value, given their tendency to produce more false alarms (Bender et al., 2010, Cohen and Faulkner, 1989, Norman and Schacter, 1997).

### 2.1 Methods

#### 2.1.1 Participants

Eighteen young (aged 21–39, mean 26.4 years, 10 females) and 18 older (aged 63–82, mean 71.9 years, 8 females) volunteers were recruited from the MRC Cognition and Brain Sciences Unit's Volunteer Panel, and were compensated financially for their time. Their inclusion was approved by the Cambridge Psychological Research Ethics Committee (reference 2005.08). Three individuals (aged 39–62, mean 57 years, 1 female) with hippocampal lesions were also tested (for additional diagnostic and lesion information, see Henson, Greve, et al., 2016). The inclusion of these patients was approved by the National Research Ethics Service, Committee East of England, Cambridge South (reference 12/EE/0190). All participants were fluent in English, had normal or corrected-to-normal vision, and provided informed consent prior to their participation. In the graphs below, “HL1” (male, aged 39) corresponds to patient “P2” in Henson, Greve, et al. (2016); “HL2” (female, aged 57) to “P5” and “HL3” (male, aged 62) to “P6”. Note that HL2 only completed the first run, and HL3 did not quite finish the second run of the experiment (see Supplementary Table 1).

#### 2.1.2 Stimuli and design

The object and scene stimuli were provided by Dr. Hugo Spiers, and were a subset of those used in Howard et al. (2011). A colour photograph of an object (e.g., cat) was superimposed either on the left or the right side of a colour photograph of a background scene (e.g., corridor). The object's vertical location was chosen to be a logical position for the object-scene composition.

246 object-scene pairings were randomly divided into two sets to create two blocks with unique stimuli. The 123 stimuli were shown twice within a block. For first presentations, half of the objects were randomly assigned to start on the right, with the other half were randomly assigned to start on the left. This assignment was reversed across participants for counterbalancing. These trials were pseudo-randomised, such that initial presentations were randomly ordered within mini-runs of four trials (unknown to participants). In each mini-run, objects were either all left, all right, or half (two) on the left and half on the right. For second presentations, 61 of the object-scene stimuli were randomly assigned to the “stay” condition and the other 62 were assigned to be in the “move” condition. This “stay”/“move” assignment was counterbalanced across participants (to produce four counterbalances in total). Second presentations were also blocked in mini-runs of four trials, in which all trials were stay trials, all were move trials, or there were half of each. The four trials of an initial presentation mini-run were then randomly intermixed with the four trials from a different mini-run of repeated presentations (i.e., involving different object-scene pairs) to produce a run of eight trials. These runs of eight trials were then concatenated, such that the stimuli used for the initial condition in one run were the stimuli used for the repeated condition in the next run but one. This procedure ensured that lag varied from a minimum of eight to a maximum of twenty-two intervening items.

#### 2.1.3 Procedure

E-Prime 2.0 was used to present the stimuli and collect the button presses. Participants were instructed to respond “new”, “stay” or “move” using their left index finger, right index finger, or right middle finger, respectively. Each object-scene pair was presented in the centre of a grey screen for 800 msec, with instructions for the button-to-finger mapping displayed at the bottom of the screen. A green fixation cross was overlaid on the centre of the screen. A button-press response was required for each trial before the next trial started. If a response was made during the object-scene presentation, then the next object-scene was presented after a random interval of 50–100 msec following stimulus offset, during which the fixation changed to a red circle to prepare participants for the next trial. If no response was detected, then a grey screen with red fixation circle remained until the response was given, which was followed by the same random interval of 50–100 msec. Prior to the first block, participants completed a practice version with 24 practice-unique object-scene stimuli.

#### 2.1.4 Standard analysis

For standard analysis of item and source memory, a “Pr” (Snodgrass & Corwin, 1988) measure of accuracy was used. For Item memory, this was the difference in the proportion of item hits minus the proportion of item false alarms (i.e., varying from −1 to 1, where 0 is chance). For Source memory, this was the number of correct minus incorrect source judgements, divided by the number of item hits. Pr was arcsine-transformed and submitted to two-sample *t*-tests with a pooled error variance. When comparing a single case against a group, this pooled variance is equivalent to Crawford and Howell (1998)'s modified *t*-test (http://www.mrc-cbu.cam.ac.uk/personal/rik.henson/personal/Henson_Singlecase_06.pdf). *P*-values are two-tailed unless reported otherwise, with alpha set as .05. One-tailed *p*-values were reported (unless *p* < .001) for comparisons of *Ds* and *Di*, where Older and HL groups were predicted to show lower values (see 2.1).

#### 2.1.5 MPT fitting

MPTs were fit using the “MPTinR” package (version 1.8.0) (Singmann & Kellen, 2013), implemented in R (version 3.2.3)(R Core Team, 2015). For each fit, 50 optimisation runs were specified, and the parameter estimates averaged across converged datasets.

MPTs are often fit to aggregate trial counts (summed across participants). We initially fit such aggregate data, with separate MPT parameters for each of the two groups under consideration. The purpose of this aggregate fit was to determine whether all parameters were estimated uniquely and to provide measures of overall model fit. We report model fit in terms of *G*^2^, Akaike's Information Criterion (AIC) and the Bayesian Information Criterion (BIC).

With aggregate data, differences between groups can be tested by the reduction in model fit when a parameter is constrained to be equal across groups, relative to when it is free to vary across groups (Riefer & Batchelder, 1988). However, this approach is not always appropriate when the groups differ dramatically in size (as in certain comparisons here), since measures of goodness of fit can be dominated by the group with more participants (higher aggregate numbers). More importantly, this aggregate approach ignores individual differences between participants (in the extreme case, ignoring the possibility that parameters differ wildly across two subsets of participants within a group, such that the parameter values from fitting the aggregate data bear no resemblance to either subset; an example of Simpson's paradox). We wanted to perform the same type of statistical analysis on the MPT parameters as is performed on conventional Pr calculations, in which participants are treated as a random effect, and the results are therefore generalisable from each sample (group) to the population from which it was drawn. Since our Paradigms 1 and 2 provided 246 and 120 trials per participant, respectively, the model parameters should be estimated sufficiently accurately (Batchelder and Riefer, 1990, Riefer and Batchelder, 1988). Given that MPT parameters reflect probabilities, we arcsine transformed them (like for Pr scores) and checked the transformed distributions were reasonably Gaussian, before submitting them to the same two-sample *t*-tests with pooled error variance across groups, as was done for Pr values.

### 2.2 Results

In the Object-Scene paradigm, there were 9 response categories: Stay trials called “Stay” (Correct source), “Move” (Incorrect source) or “New” (misses); Move trials called “Stay” (Incorrect source), “Move” (Correct source) or “New” (misses); and New trials called “Stay” (False Alarm), “Move” (False Alarm) or “New” (Correct Rejection). To examine age effects, we compared a group of *N* = 18 healthy Young versus *N* = 18 healthy Older people; to examine effects of hippocampal lesions, we compared the *N* = 18 Older group to *N* = 3 patients.

The full MPT Item-Source model is shown in Fig. 2A, while that for the Source-Item model is shown in Fig. 3A. We first tested whether all parameters (*Dn*, *Di, Ds*, *Gi*, *Gs*) were required. The data were insufficient to estimate *Dn* uniquely, i.e., the fit was equivalent when *Dn* = 0. Therefore *Dn* was removed in all subsequent analyses, corresponding to a “1HT” model (Snodgrass & Corwin, 1988). This left 12 independent response categories, fit by 8 parameters.

#### 2.2.1 Effects of age

##### 2.2.1.1 Standard Pr analysis

**Fig. 5:**
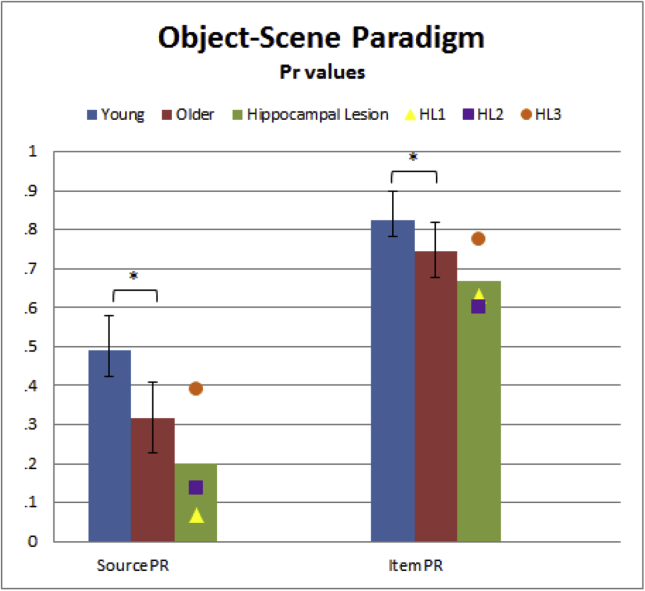
Source and Item memory Pr values from Object-Scene Paradigm for Young, Older, and Hippocampal Lesion (HL) groups. Group differences are marked: * = significant at *p* < .05 two-tailed; + = significant *p* < .05 one-tailed for a directional hypothesis. Error bars represent two-tailed 95% confidence intervals from a pooled error term (after inverse arcsine transformation), while individual scores are provided for the HL group.

In the Object-Scene paradigm, a standard analysis of Source memory showed significantly smaller mean Pr value in the Older group (Pr = .318) than Young group (Pr = .492), *t*(34) = 3.66, *p* < .001. Item memory was also significantly smaller in Older (Pr = .744) than Young (Pr = .825) groups, *t*(34) = 2.44, *p* = .010, one-tailed. Pr values for each group are provided in Fig. 5.

##### 2.2.1.2 MPT analysis

**Fig. 6:**
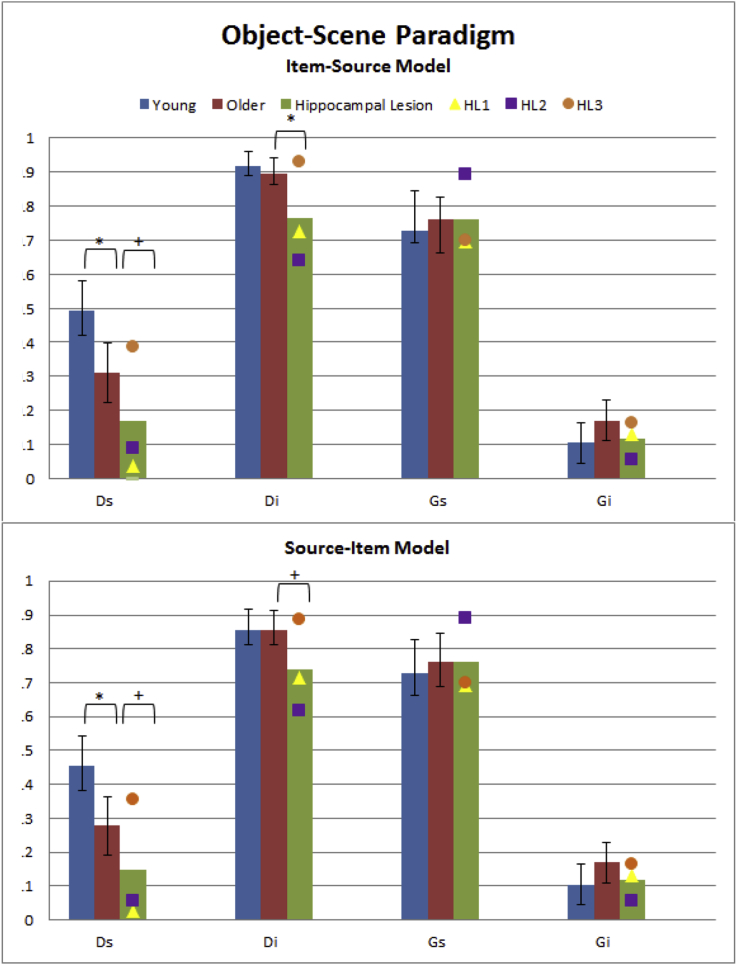
Individual parameter estimates averaged per group for MPT Item-Source and Source-Item models on the Object-Scene paradigm for Young, Older, and Hippocampal Lesion (HL) groups. Note that the *Gs* and *Gi* are constrained to be identical across the two models (see Supplementary Material). See Fig. 5 legend for more details.

As expected, the Item-Source model and Source-Item model fit the aggregate Young and Older data equally well, *G*^2^(4) = 82.0, AIC = 98.0, BIC = 160.2. Despite equivalent overall model fit, some of the parameters differed across models (see Supplementary Proof), as can be seen from the average parameter estimates after fitting individual participants shown in Fig. 6. Raw trial counts are shown in Supplementary Table 1.

The mean source memory parameter (*Ds*) was slightly larger in the Item-Source than Source-Item model, but it was significantly lower in the Older group than Younger group according to both models [*t*(34) = 3.80, *p* < .001, and *t*(34) = 3.74, *p* < .001, respectively]. Unlike the standard Pr analysis, the mean Item memory parameter (*Di*) was not reliably different between the Older and Young groups according to either model [Item-Source: *t*(34) = 1.10, *p* = .140, one-tailed; Source-Item: *t*(34) = .05, *p* = .480, one-tailed].

Within this paradigm, the remaining two MPT parameters – the probability of guessing the source location had not changed (*Gs*) and the probability of guessing an item was studied (*Gi*) – are necessarily identical across the two MPT models. The *Gs* parameter was around .75 on average across groups, indicating a tendency to guess the source location had stayed the same between initial and repeated presentations, but there was no evidence that this value differed between groups [*t*(34) = .51 *p* = .613, two-tailed]. There was a suggestion, on the other hand, that the Older group showed an increased tendency to guess that items were studied [about .18, relative to .10 in the Young, *t*(34) = 1.84, *p* = .074, two-tailed].

#### 2.2.2 Effects of hippocampal lesions

##### 2.2.2.1 Standard Pr analysis

Any reduction in Source (Pr = .199) or Item (Pr = .669) memory for the Hippocampal group, relative to the Older group (Pr = .318 and Pr = .744, respectively), failed to reach significance using standard Pr scoring, *t*(19) = 1.65, *p* = .058, one-tailed, and *t*(19) = 1.23, *p* = .117, one-tailed, respectively. The lack of significant effects is likely to reflect the large variability within the patient group (see Fig. 5). Therefore each patient was also compared to the Older group separately, but in none of the three cases did the difference reach significance for Item memory, *t*(17) = 1.11, *p* = .141, *t*(17) = 1.35, *p* = .098, *t*(17) = −.24, *p* = .593 (HL1–HL3, respectively, all one-tailed), and only in one case (HL2) did it reach significance for Source memory, *t*(17) = 2.26, *p* = .019, one-tailed [*t*(17) = 1.69, *p* = .055 and *t*(17) = −.69, *p* = .750, for HL1 and HL3, respectively, all one-tailed].

##### 2.2.2.2 MPT analysis

The fit indices for both MPT models to the aggregate Older and HL data were *G*^2^(4) = 6811.9, AIC = 52.6, BIC = 110.3. Unlike the standard Pr analysis, the MPT estimates for individual model fits showed reductions that were significant in both Source and Item memory in the HL group, according to both the Item-Source [*Ds*: *t*(19) = 1.83, *p* = .042; *Di*, *t*(19) = 2.30, *p* = .016; both one-tailed] and Source-Item models (*Ds*: *t*(19) = 1.80, *p* = .044, *Di*: *t*(19) = 1.83, *p* = .042; both one-tailed). There was no significant difference between Hippocampal and Older groups in either of the guessing parameters [*Gs*: *t*(19) = .03, *p* = .975; *Gi*: *t*(19) = .93, *p* = .362].

As in the Pr analysis, each patient was also compared to the Older group separately. According to the Item-Source model, the *Di* parameter was significantly smaller in two patients (HL1 and HL2), *t*(17) = 2.12, *p* = .024, *t*(17) = 2.91, *p* = .005, but not the third (HL3), *t*(17) = −.39, *p* = .649, and the same was true for the *Ds* parameter, *t*(17) = 2.39, *p* = .014 (HL1), *t*(17) = 1.93, *p* = .035 (HL2), but not the third, *t*(17) = −.67, *p* = .744 (all one-tailed).

According to the Source-Item model, the *Di* parameter was significantly smaller in one patient (HL2), *t*(17) = 2.25, *p* = .019, but not the other two (HL1 and HL3), *t*(17) = 1.46, *p* = .082, *t*(17) = −.25, *p* = .597 (all one-tailed). As in the Item-Source model, the reduction in the *Ds* parameter was significant in the same two patients, *t*(17) = 2.32, *p* = .017 (HL1) and *t*(17) = 2.04, *p* = .029 (HL2), but not in HL3, *t*(17) = −.75, *p* = .768 (all one-tailed). *Gs* and *Gi* parameters, as mentioned above, are identical for the two models and there were no significant differences between the Older group and the Hippocampal Lesion group [*Gs*: *t*(17) = .61, *p* = .549, *t*(17) = 1.12, *p* = .277, *t*(17) = .57, *p* = .577; *Gi*: *t*(17) = .41, *p* = .685, *t*(17) = 1.16, *p* = .261, *t*(17) = .07, *p* = .949, for HL1–HL3, respectively].

### 2.3 Discussion

A standard Pr analysis provided evidence that age significantly impairs both Source and Item memory, whereas the MPT analyses provided evidence that age significantly impairs Source memory, but no evidence that it affects Item memory. This contrasting pattern of results does not seem to reflect a generally reduced sensitivity of MPT analyses relative to standard analysis, since the standard analysis provided little evidence that hippocampal lesions affect either Source or Item memory, where one would expect clear evidence of amnesia: There was no significant difference when comparing the group values of Pr, and the difference was significant in only one of the six comparisons of Item and Source memory when testing the three patients individually. Both MPT analyses, on the other hand, provided stronger evidence that hippocampal lesions impair both Source and Item memory, both when comparing the groups, and for four of the six individual comparisons (with one of the three patients, HL3, consistently being the exception).

This variable pattern of significant effects between standard and MPT analyses illustrates how different conclusions could emerge from using different methods of analysing source monitoring tasks. One might wonder whether one difference between the Pr and MPT results for Item memory is that the Pr measure includes an explicit adjustment for false alarms, whereas the MPT parameter *Di* is a “pure” estimate of remembering a studied item. However, this difference is more apparent than real, since the MPT implicitly takes into account false alarms via the *Gi* parameter (indeed, *Gi* tended to be higher in the Older than Young group). Because the MPT approach estimates *Di* and *Gi* simultaneously, *Di* already includes an “adjustment” for *Gi* (i.e., *Di* is not simply the raw hit rate, as evident from Figs. 2A and 3A). Indeed, the estimation of additional parameters like the *Gi* and *Gs* guessing parameters is another advantage of the MPT approach. In sum, though the Pr index is also derived from a 1HT model of simple yes/no recognition memory tasks (Snodgrass & Corwin, 1988), and so has an equivalent MPT for that task, MPTs represent a more complete model of more complex source monitoring tasks like the present one.

As expected, both MPT analyses confirmed the Older group to have lower values of *Ds* than the Young Group, consistent with impaired source retrieval/recollection with age (Hashtroudi et al., 1989, Koen and Yonelinas, 2014, McIntyre and Craik, 1987, Spencer and Raz, 1995), but provided no evidence that *Di* differed, consistent with claims that item memory/familiarity less affected by ageing (Koen and Yonelinas, 2014, Naveh-Benjamin, 2000). We also confirmed our expectation for lower values of *Ds* in the HL group than Older (control) group, consistent with claims that the Hippocampus is necessary for recollection (Yonelinas, 2002) (perhaps in conjunction with Prefrontal cortex), but also found lower values of *Di*, which is not consistent with claims that regions like Perirhinal cortex are sufficient for item memory (Norman & O'Reilly, 2003). Nonetheless, we refrain from further interpretation until after the results of Paradigm 2.

Another important advantage of MPT analyses is that they make explicit the assumptions behind how task performance is supported by underlying psychological factors, such as the nature of memory systems or processes described in the Introduction. For Paradigm 1, the Item-Source and Source-Item models (Figs. 2A vs 3A) fit the data equally well, and while producing different quantitative estimates of *Ds* and *Di*, they did not produce different qualitative patterns of significance when comparing the various groups. In the richer dataset described next, however, the two models fit the data differently and produced different patterns of significance.

## 3 Paradigm 2: Object-Location

**Fig. 7:**
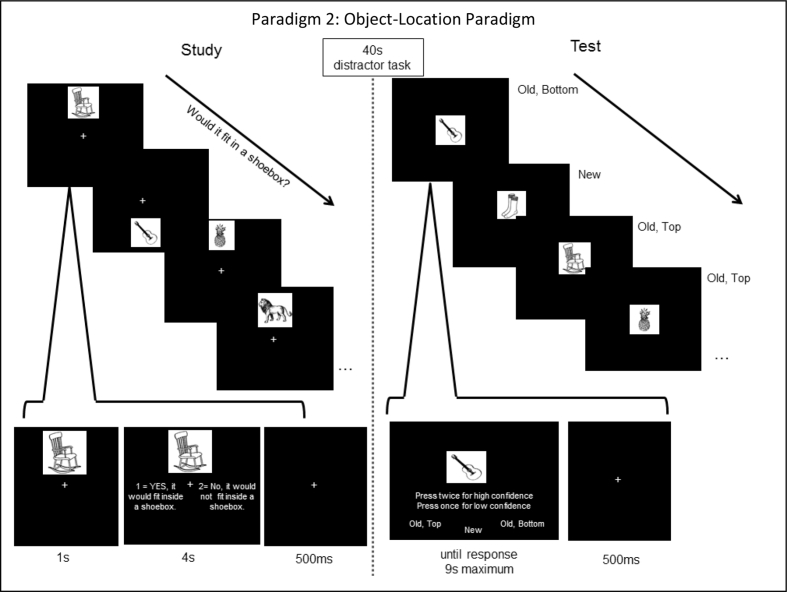
Paradigm 2: Object Location Paradigm. This was a study-test blocked source monitoring paradigm, in which items were studied at top or bottom locations while making “yes”/”no” judgement as to whether they would fit in a shoebox. A 40 s (sec) distractor task of counting backwards occurred between study and test blocks. In test blocks, participants reported whether items were “new” or had appeared in “top” or “bottom” locations, pressing the response key twice if highly confident.

Paradigm 2 is shown in Fig. 7. This was a more conventional source monitoring paradigm, consisting of study and test blocks. In study blocks, objects were presented either above or below the central fixation point, while in test blocks, they were presented centrally at fixation, intermixed with unstudied objects, for which participants made a “new”, “top” or “bottom” response. To fit the MPT models, S1 was defined as “bottom” and S2 was defined as “top”, such that *Gs* was the probability of guessing “bottom”, and 1-*Gs* was the probability of guessing “top” (again, these assignments are arbitrary, and do not affect the conclusions). Participants also provided confidence ratings, which enabled more complex MPTs than Paradigm 1 (See also Fig. 2A vs 2B and Fig. 3A vs 3B).

The high vs low confidence ratings resulted in 18 response categories, which enabled four new parameters to be estimated. These included the *Dn* parameter, which was now needed (unlike in Paradigm 1) to model high confidence correct rejections. This parameter can capture situations like the use of meta-memory, where a participant rejects a new item as studied because they are confident they would have remembered it (Johnson et al., 1993). The other three parameters were: 1) a *Df* parameter, reflecting the probability of high confidence false alarms (e.g., false recollection); 2) a *Dh* parameter, reflecting the probability of high confidence source hits (e.g., vivid recollection); and 3) a *Dm* parameter, reflecting the probability of high confidence misses (e.g., owing to missed encoding of a Study trial) (Snodgrass & Corwin, 1988).

This paradigm was run on a Young and Older group (*N* = 12 each), and *N* = 105 adults with mild memory problems (MMP group). As for Paradigm 1, we expected *Ds* and, if at all, *Di*, to decrease with age, particularly in those reporting everyday memory problems. As described in the Introduction (based on prior studies), we also expected *Dh* to similarly decrease, but for *Df* and *Dm* parameters to increase with age and in those reporting everyday memory problems. We had no directional predictions about how *Gs* and *Gi* might differ.

### 3.1 Methods

#### 3.1.1 Participants

Twelve young (aged 20–51, mean 28 years, 10 females) and 12 older (aged 55–80, mean 71 years, 6 females) volunteers were recruited from the MRC Cognition and Brain Sciences Unit's Volunteer Panel. Their inclusion was approved by Cambridge Psychological Research Ethics Committee (reference 2005.08). An additional 105 older individuals (aged 55–81, mean 66 years, 44 females), who all reported mild memory problems (henceforth the MMP group), were recruited as part of a separate study sponsored by Glaxo-Smith Kline (GSK). These individuals completed the Wechsler Memory Scale, Fourth Edition (WMS-IV) (Wechsler, 2010) and the Mini-Mental State Examination (Folstein, Folstein, & McHugh, 1975). Participants were selected for inclusion in the present study when their WMS-IV Logical Memory score was either below 24 on the immediate test or below 22 on the delayed test, indicating memory problems, but their MMSE score was above 25, indicating no clinically significant impairment of general cognitive abilities. Their inclusion was approved by the National Research Ethics Service, Committee East of England–Cambridge South (reference 12/EE/0382). All participants were fluent in English, had normal or corrected-to-normal vision, provided informed consent prior to their participation and were compensated financially for their time.

#### 3.1.2 Stimuli and design

480 black line-drawings of objects on a white background were used to create 8 blocks of a study-test design. The stimuli were from the International Picture Naming Project (Szekely et al., 2004; http://crl.ucsd.edu/∼aszekely/ipnp/). Items were divided equally into 8 sets of 60 items. 40 items in each set were randomly selected to be the study items, half of which were assigned to the “top” condition and half to the “bottom” condition, with the constraint that item locations were not wholly congruent or incongruent with their typical placement in the real-world, e.g., a bird, hat, and plane were not always assigned to the “top”. The order of study trials was randomised. The remaining 20 items from a set formed the “new” items in the test section, which were randomly intermixed with studied items during the subsequent test block.

The Older group completed 2 sets of stimulus sets 1–4, either 1 and 2 or 3 and 4, and the young group completed sets 5 and 6. The data from the MMP group are from the two sets completed in their initial baseline visit, for which the assignment of stimulus set 1–8 was counterbalanced. This allowed us to test for differences in performance across stimulus sets (on initial visit of MMP group), for which there was no evidence that the sets differed, *F*(7,98) = 1.38, *p* = .222.

#### 3.1.3 Procedure

E-Prime 2.0 was used to present the stimuli and collect button presses. Each participant completed 2 study blocks and 2 test blocks in a study-test-study-test procedure. Study and test blocks were separated by a distractor task, during which participants counted backwards in 3's from a random starting point for 40 sec. For the study blocks, participants were instructed to decide whether each object depicted would fit in a shoebox, using left and right index fingers on a button-box. Before starting the study sections participants were made aware via instruction and practice that their memory for each object's location would be explicitly tested. Each trial began with a white fixation cross on a black background presented in the centre of the screen for 500 msec. The fixation cross remained on the screen while each study item was presented in their assigned “top” or “bottom” location. Items were displayed for a total of 5000 msec, regardless of response, with instructions for the button-to-finger mappings at either side of the fixation cross appearing after 1000 msec.

For test blocks, participants made their “top”, “bottom” or “new” response using top left, top right, or lower middle button with any fingers, though most used left index finger, right index finger, and thumbs, respectively for the Older and Young groups. For the MMP group, mappings were right index finger, right middle finger, and left index finger, respectively, on three buttons in the same row. To indicate confidence, participants pressed the button twice if they were confident, and only once otherwise. Each trial began with a white fixation cross on a black background presented in the centre of the screen for 500 msec. The test items were presented in the centre of the screen with instructions for the button-to-finger mappings and how to report confidence at the bottom of the screen. For the Young and Older groups, items remained on the screen until a response was made, or for a maximum of 9000 msec. For the MMP group, items remained on the screen for 3500 msec regardless of response, and were followed by a random interval between 410 and 600 msec. This was because the MMP group were tested with concurrent electroencephalography (EEG) (not reported here), for which a fixed SOA was deemed preferable. Trials with no response, or where two different response buttons were pressed (mixed response), were discarded. Prior to the first block, participants completed two practice study-test blocks with 6 practice-unique items.

#### 3.1.4 MPT fitting

The Item-Source and Source-Item MPT models, extrapolated to account for confidence data (Figs. 2B and 3B), were fit in the same manner as for Paradigm 1. Unlike Paradigm 1, the *Dn* parameter could be estimated uniquely (corresponding to a 2HT model). Moreover, the Item-Source and Source-Item models differed in their ability to fit the data, and only the *Dn* parameter was constrained to be identical across models (see Supplementary Proof). There were 30 independent response categories, fit by 16 parameters.

As detailed above, we report one-tailed *p*-values for tests on the discriminability (D) parameters, based on the predicted decreases in *Ds*, *Di* and *Dh* across groups, and increases in *Df* and *Dm*, but two-tailed *p*-values for the tests of the guessing (G) parameters, for which we had no clear predictions.

### 3.2 Results

To examine age effects, we compared a group of *N* = 12 healthy Young versus *N* = 12 healthy Older people; to examine effects of possible memory disorders in ageing, we compared the *N* = 12 healthy Older people to *N* = 105 Older people with memory problems (MMP group) (see 3.1).

#### 3.2.1 Effects of age

##### 3.2.1.1 Standard Pr analysis

**Fig. 8:**
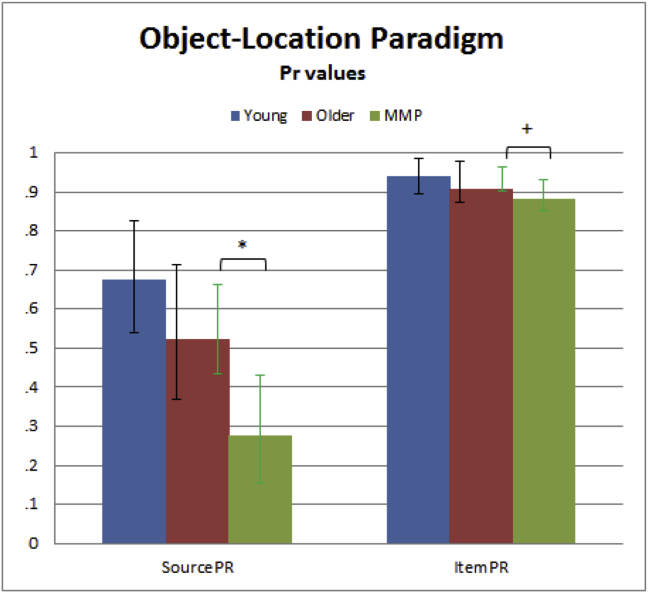
Object-Location Paradigm Source and Item memory Pr values for Young, Older, and Mild Memory Problem (MMP) groups. Group differences are marked * = significant at *p* < .05 two-tailed; + = significant *p* < .05 one-tailed for directional hypothesis. Error bars represent two-tailed 95% confidence intervals (after inverse arcsine transformation), after pooling error for a specific comparison of two groups: black bars come from comparison of Young and Older groups while green bars come from comparison of Older and MMP groups.

A standard Pr analysis of data from the Object-Location data failed to reveal differences between Young and Older groups that reached significance in either Source memory (Pr = .675 and Pr = .523, respectively), *t*(22) = 1.55, *p* = .068, one-tailed, or Item memory (Pr = .940 and Pr = .908, respectively), *t*(22) = .50, *p* = .311, one-tailed. See Fig. 8.

##### 3.2.1.2 MPT analysis

**Fig. 9:**
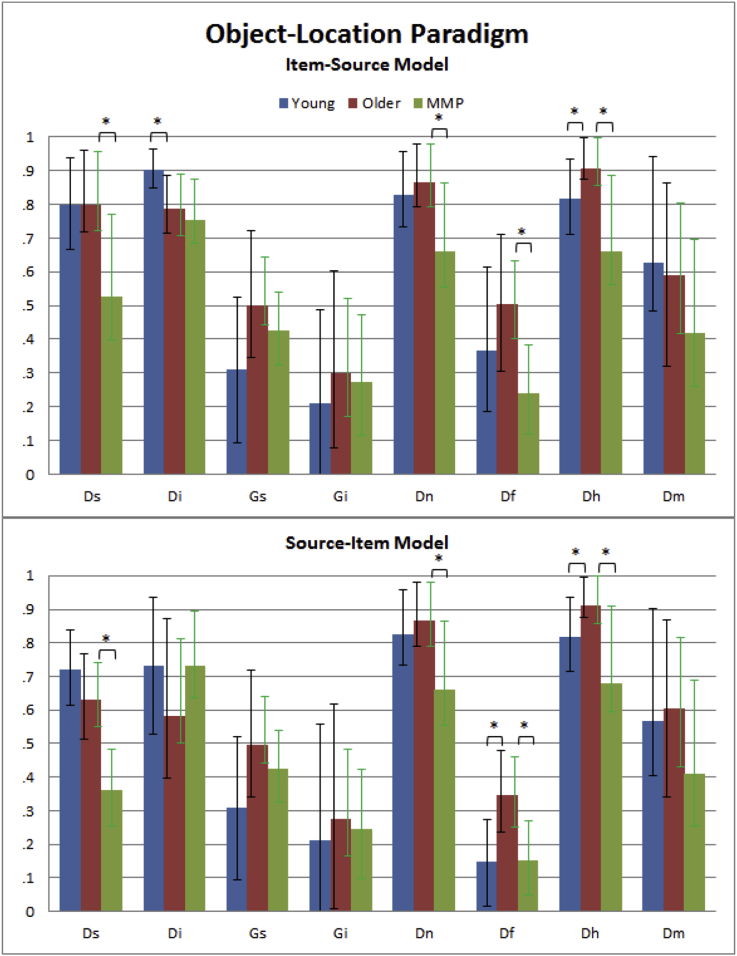
Individual parameter estimates averaged per group for MPT Item-Source and Source-Item models applied to the Object-Location paradigm for Young, Older, and Mild Memory Problems (MMP). See Fig. 8 legend for more details.

The full MPT for Item-Source model and Source-Item models are shown in Figs. 2B and 3B, respectively. Interestingly, the Source-Item model fit the aggregate data better, *G*^2^(14) = 42.51, AIC = 74.51, BIC = 169.87, than the Item-Source model, *G*^2^(14) = 126.87, AIC = 158.87; BIC = 254.23. The average of the individual parameter estimates for the Item-Source and Source-Item MPTs and for each of the three groups (Young, Older and MMP) are shown in Fig. 9. The raw trial counts are in Supplementary Table 2.

Unlike the standard Pr analysis, the Item-Source MPT revealed an effect of age on the *Di* parameter, with the Older group having lower estimates than the Young, *t*(22) = 2.55, *p* = .009, one-tailed, though the same was not true for the Source-Item MPT, *t*(22) = .77, *p* = .225, one-tailed. Unlike Paradigm 1, neither MPT model showed any evidence that age affected the *Ds* parameter, *t*(22) = −.52, *p* = .696, one-tailed, and *t*(22) = 1.22, *p* = .118, one-tailed. Neither guessing parameter, *Gi* or *Gs*, showed an effect of age in either the Item-Source model [*Gi*, *t*(22) = .84, *p* = .411, *Gs*, *t*(22) = 1.94, *p* = .066] or Source-Item model [*Gi*, *t*(22) = .37, *p* = .712, *Gs*, *t*(22) = 1.92, *p* = .068], though the trend in the *Gs* parameter in both models reflected a bias towards guessing the “top” location in the Young group.

The new *Dn* parameter, which is identical across models, did not decrease with age, *t*(22) = −.69, *p* = .751, one-tailed, and nor did the new parameter *Dm* increase with age for either the Item-Source, *t*(22) = −.87, *p* = .803, one-tailed, or Source-Item model, *t*(22) = −.34, *p* = .631, one-tailed. More interestingly, the new parameter for high confidence false alarms, *Df*, was significantly higher in the Older group, as expected, at least according to the Source-Item model, *t*(22) = 2.90, *p* = .004, one-tailed, though not Item-Source model, *t*(22) = .89, *p* = .192, one-tailed. The *Dh* parameter for high confidence source hits was also higher in the Older group – the opposite of the predicted decrease with age – according to both Item-Source and Source-Item models, *t*(22) = −2.24, *p* = .036, and *t*(22) = −2.27, *p* = .033, respectively.

#### 3.2.2 Effects of mild memory problems

##### 3.2.2.1 Standard Pr analysis

In a standard Pr analysis, the MMP group showed significantly worse Source memory (Pr = .277), *t*(115) = 3.39, *p* < .001, and Item memory (Pr = .883), *t*(115) = 1.95, *p* = .027, one-tailed, than the Older group above.

##### 3.2.2.2 MPT analysis

Again, the Source-Item model fit the data better, *G*^2^(14) = 297.45, AIC = 329.45, BIC = 449.97, than the Item-Source model, *G*^2^(14) = 1190.48, AIC = 1222.48. Turning to results with individual parameter estimates, both Item-Source and Source-Item models showed significantly worse Source memory (*Ds*) in the MMP group, *t*(115) = 2.83, *p* < .003, one-tailed, and *t*(115) = 4.36, *p* < .001, respectively, but no significant difference in Item memory [*Di*, *t*(115) = .35, *p* = .363, one-tailed, and *t*(115) = −1.29, *p* = .902, one-tailed, respectively]. Both models were also in agreement that there was no evidence of differences in item guessing [*Gi*, *t*(115) < .68, *p* > .498 and *Gs*, *t*(115) < 1.77, *p* > .079; all one-tailed], or missed encoding [*Dm*, *t*(115) = 1.09, *p* = .278 and *t*(115) = 1.24, *p* = .216, respectively]. However, both models showed evidence for lower rates of high confidence false alarms [*Df*, *t*(115) = 3.58, *p* < .001 and *t*(115) = 3.06, *p* = .001, respectively], high confidence source hits [*Dh*, *t*(115) = 2.87, *p* = .002, one-tailed, and *t*(115) = 2.60, *p* = .005, one-tailed, respectively] and detection of new items [*Dn*, *t*(115) = 2.37, *p* = .010, one-tailed] in the MMP group.

### 3.3 Discussion

Different patterns of significance were again found depending on the type of analysis used in this Object-Location paradigm. For example, the standard Pr analysis did not detect any age effects on Item or Source memory, yet the Item-Source MPT, but not Source-Item MPT, showed an age-related reduction in the Item memory parameter, *Di*. In terms of the effects of mild memory problems, the standard Pr analysis suggested that this group has problems in both Source and Item memory, whereas both MPT models only showed a problem in Source Memory (*Ds*).

Furthermore, the confidence ratings available in this paradigm enabled the MPT models to surpass traditional Pr analyses and estimate a number of other group differences. The parameters capturing the probability of detecting new items (*Dn*) and of missing the encoding of old items (*Dm*) did not differ between Young and Older groups, but that capturing the probability of high confidence false alarms (*Df*) was higher in the Older group, at least according to the Source-Item MPT model. The former findings suggest that age did not affect the use of meta-memory strategies for rejecting unstudied items, or affect the number of trials that failed to be encoded at Study, e.g., owing to lapses in attention. While the latter finding (*Df*) might suggest that age increases the incidence of false recollection (Dodson et al., 2007), both MPT models also suggested that age increases the probability of higher confidence source hits (*Dh*), contrary to expectations. The higher values for both *Dh* and *Df* suggests that the Older group were more confident in general. More interesting is the observation that this greater confidence occurred despite no evidence of greater accuracy in Source memory (*Ds*), suggesting that the Older group were over-confident in their source memory abilities.

This over-confidence is not a feature of all older people, since the group with mild memory problems (MMP group) showed values for *Df* closer to the Young group, and values of *Dh* even lower than the Young group (Fig. 9). The MMP group also had lower values of Source memory (*Ds*) and worse detection of New items (*Dn*) (but no evidence that they were worse at detecting studied items, *Di*). This suggests that Source Memory was generally impaired in the MMP group, and perhaps because members of that group were aware of memory problems, they rarely used high-confidence responses, explaining the lower values for *Dh* and *Df*.

One caveat is that different stimulus sets were used for different groups. While it remains possible that the group differences we found reflect stimulus differences, we think this is unlikely because we found no evidence for effect of stimulus set when analysing the fully-counterbalanced MMP group (see Stimuli and design section). Another caveat concerns differences in the test procedure, which used a fixed SOA for the MMP group, but a self-paced design for Older (and Young) group. Further investigation would be needed to see whether this affected the results. It should also be noted that the Older group may not be typical of the older population, since they volunteered for psychological research, and therefore may have been above-average in terms of their motivation and general cognitive health. Nonetheless, it is unclear how either of these factors would explain the different patterns of results across the Pr and two MPT analyses.

When comparing the two MPT models, it is interesting that the Source-Item model provided a better fit than the Item-Source model when applied to both the Young/Older and Older/MMP aggregate data. Furthermore, when comparing parameters from individual fits to Young and Older groups, the results from the two MPT models differed on two parameters (*Di* and *Df*), thus influencing the conclusions one would draw about the effects of age. As noted in the Introduction, these two models differ in their interpretation of the *Di* parameter. According to the Item-Source model, age reduces the probability of remembering an item, with or without corresponding source information, whereas according to the Source-Item model, age does not affect the probability of remembering an item, provided source information is not recalled. Furthermore, the potentially important conclusion that (healthy) ageing affects false recollection was only provided by the Source-Item model. These differences between MPT models show that, without considering the theoretical basis of the task (e.g., whether performance depends on one or more than one source of information), one cannot make general statements about the effects of a variable like age.

## 4 General discussion

Across two different source monitoring paradigms, and six independent groups of participants, the significance of the effects of age, of hippocampal lesions and of mild memory problems on Source and Item memory depended on the manner in which the data were scored. This underlines the importance of considering the assumptions underlying the scoring method. Specifically, analysis using Multinomial Processing Tree (MPT) models produced different patterns of significant effects from analysis using standard accuracy estimates (Pr), while two different MPT models also sometimes produced divergent results. We summarise these differences below, before considering their implications.

### 4.1 Summary of results

The Object-Scene paradigm was a continuous source monitoring paradigm. It revealed an effect of age on Source memory in both Pr analysis and MPT analyses. However, whether age affected Item memory depended on the analysis: traditional Pr analysis showed significant effects, while neither of the two MPT models did. The MPT models also provided estimates of additional guessing parameters, namely the probability of guessing an item as studied (*Gi*), and the probability of guessing a particular source (*Gs*). The *Gi* parameter, but not *Gs* parameter, was higher in the Older group. The lack of an effect of age on item memory (*Di*), coupled with a significant effect on guessing (*Gi*), suggests that item memory signals (e.g., familiarity) are not affected by age, while there is an age-related bias to call items “old”, consistent with prior claims of increased false alarms with ageing (Bender et al., 2010, Cohen and Faulkner, 1989, Dodson et al., 2007, Norman and Schacter, 1997). When comparing the Older group against a group of three patients with amnesia following hippocampal lesions (HL group), significant reductions in both Source and Item memory were found, but only in the MPT analysis, not the Pr analysis (and when each patient was compared separately, a greater number of individual comparisons were significant with MPT analysis than Pr analysis).

The Object-Location paradigm was a more conventional blocked study-test source monitoring paradigm. This time, the standard Pr analysis did not reveal any difference between Young and Older groups on either Item or Source memory, but the MPT models did (i.e., the converse pattern of significant findings for Pr versus MPT to that found in the Object-Scene paradigm). Furthermore, the two MPT models now also produced different results. This was because the additional response categories furnished by the confidence ratings in the Object-Location paradigm allowed more complex MPTs to be fit, with several interesting additional parameters. These extra parameters also meant that the Item-Source and Source-Item models were no longer isomorphic. Interestingly, all three metrics of model fit showed the Source-Item model fit the aggregate data better than the Item-Source model. Furthermore, the parameter estimates from fitting individual data revealed an effect of age on Item memory (*Di*) according to the Item-Source model, but not according to the Source-Item model. Thus only the Source-Item model produced consistent (lack of) effect of age on item memory across the two paradigms.

Neither MPT model showed an effect of age on the basic source memory parameter (*Ds*) in the Object-Location paradigm, but did reveal an age effect on two related source memory parameters associated with high-confidence responses: Both MPT models showed that the Older group had an increased probability of high confidence source hits (*Dh*), while the Source-Item MPT additionally revealed an increased probability of high confidence source false alarms (*Df*). These results, at least from the Source-Item MPT, suggest that the Older group were more confident of their Source memory, even though not necessarily more accurate. Interestingly, however, there was no evidence that this group was more confident when calling studied items new (misses), as captured by the *Dm* parameter, owing to missed encoding of study trials for example.

Comparison of the Older group against a group of similarly aged individuals with mild memory problems (MMP group) revealed that the MMP group was impaired in both Source and Item memory according to Pr analysis, but only in Source memory according to both MPT models. Both MPT models also suggested that the MMP group showed smaller *Dh* and *Df* parameters, reflecting a reduced incidence of high confidence results, which may be caused by the MMP group's awareness of their memory problems. This may also explain why the MMP group showed a smaller *Dn* parameter, with fewer high confidence correct rejections of unstudied items. Importantly, the reduced *Ds* parameter showed that the MMP group were impaired in basic source memory, regardless of their lack of confidence.

### 4.2 Advantages of MPT models

The theoretical advantages of using MPT models for paradigms like source monitoring have been described before (e.g., Batchelder and Riefer, 1990, Dodson et al., 2007, Murnane and Bayen, 1996, Simons et al., 2004), and the differences in our empirical results for standard (non-MPT) versus MPT analyses reinforce the importance of optimising scoring methods. One reason why MPTs have not always been adopted is that they require numerical fitting, but software for doing so is increasingly available, such as the free MPTinR package we used in R. Another reason why MPTs are not used may be that they are normally fit to aggregate data (summed over participants), in order to maximise the response counts for estimating probabilities efficiently. Differences in parameters are then inferred by comparing model fits with and without those parameters equated across groups. The problem with the aggregate approach is that it ignores individual differences in parameters (as in the hippocampal lesion group here), nor does it cope well with different group sizes (as when comparing the 12 healthy controls versus the 105 members of the MMP group here). We took the less typical approach of fitting each individual's data, and then comparing the mean parameter estimates across groups, relative to the variability in those estimates, as is more typical in psychological experiments (and as typically done for Pr analyses). Given that the parameters reflect probabilities, care must be taken over their bounded distribution, but visual inspection suggested that an arcsine transform rendered the present distributions sufficiently Gaussian for parametric statistics. This approach does rely on there being sufficient numbers of responses per participant, for which there are guidelines (Riefer & Batchelder, 1988), or which could be checked by generating data from candidate MPTs and refitting them.

There are recent advances in the fitting of MPT models that address this issue of participant-level versus group-level inference (and even item-level inference), such as the use of continuous beta distributions for modelling participant variance and hierarchical Bayesian techniques (Klauer, 2010, Matzke et al., 2015; Smith & Batchelder, 2010). Some of these techniques have been applied to source memory data (Arnold, Bayen, Kuhlmann, & Vaterrodt, 2013). There are also advances in metrics for model fit, such as the Fisher Information Approximation (Singmann & Kellen, 2013) and Minimum Description Length (Kellen et al., 2013). However, our focus here is not on the latest developments in MPT fitting, but rather on the structure of the MPTs themselves, and how these relate to different psychological theories. A strength of MPT models is that they make explicit the assumptions underlying the scoring method. Indeed, it was only by thinking about MPT structures that we came up with the present Source-Item MPT, as an alternative to the more commonly used Item-Source MPT. These two models produce different numerical (and statistical) estimates for some of their parameters, which in turn leads to different interpretations. As described in the Introduction, the traditional Item-Source model (e.g., Batchelder and Riefer, 1990, Dodson et al., 2007, Simons et al., 2004) is consistent with a single memory representation that can vary in quality, with sufficient information to support both Item and Source memory, or else support Item memory only. The Source-Item model, on the other hand, allows for the possibility that one memory representation supports both Item and Source memory, whereas another representation supports Item memory. This is consistent with neuropsychological and neuroimaging data, which suggest that some brain regions (such as perirhinal cortex) only support Item memory (e.g., via a global match of perceptual information), while other regions (such as hippocampus) support Source memory (e.g., via pattern completion of episodic information). While the present data cannot conclusively support one MPT model over the other, we found that the Source-Item model fits the Object-Location data better, for both group comparisons, and provides more consistent effects of age on the *Di* and *Df* parameters. This is interesting because most MPT papers on Source Monitoring have used the “Item-Source” model, and we are not aware of any papers that have considered the alternative “Source-Item” model. The Source-Item model therefore represents an important novel contribution of this study, which deserves consideration in future MPT modelling of source monitoring paradigms.

### 4.3 Effects of age, hippocampal lesions and memory problems

While we emphasise that the conclusions one would draw from the present data depend on the scoring method, we believe that the Source-Item model is more consistent with brain data from the literature. Moreover, when comparing fit metrics of the two models with aggregate data from the same groups on Paradigm 2, the Source-Item model has lower, more favourable metrics than the more traditional Item-Source MPT model, indicating the Source-Item model better accounts for the data in both the ageing and MMP analyses. The individual parameter estimates from the Source-Item model also produced the pattern of significant results that is most in keeping with current psychological theories. This includes the claim that healthy ageing is associated primarily with a reduction in Source memory rather than Item memory (Cohen and Faulkner, 1989, McIntyre and Craik, 1987, Schacter et al., 1991, Spencer and Raz, 1995). This was the pattern found in the Object-Scene experiment, and may reflect greater age-related atrophy of hippocampus than perirhinal regions (Henson, Campbell, et al., 2016). It was not the pattern found in the Object-Location experiment (where no effect of age was found on either Item or Source memory), but one reason for this may be the greater number of stimuli in the Object-Scene experiment, which may have increased interference between episodic representations to a sufficient degree to reveal an age-related impairment. Nonetheless, the confidence judgements in the Object-Location experiment were sufficient to indicate another effect of ageing that has been observed previously, namely an increased tendency for high confident false alarms (consistent with the concept of age-related increases in “false recognition”, Dodson et al., 2007). In fact, this increased confidence was found for both incorrect and correct source responses, suggesting that the confidence was misplaced, in that it did not reflect greater Source memory accuracy than the Young group.

This difference between healthy Older and Young groups contrasts with the difference between the healthy Older group and the group with Mild Memory Problems (MMP). The MMP group did not show evidence of over-confidence, but importantly did show a reduction in Source memory, compared to their age-matched “controls”. Nonetheless, one should keep in mind that the healthy Older group may not be a true “control” group for ageing, since it may consist of atypically healthy and motivated older individuals. Indeed, the over-confidence, combined with a lack of Source memory impairment (provided there are not too many interfering trials), may be specific to such “super-normal” elders.

The MMP group also showed a reduction in the *Dn* parameter. This parameter captures a process of active rejection of new items. This process is assumed to be based on metacognitive inference, such as inferring an unstudied item is new on the basis of some unusual feature of it, e.g., “I definitely would have remembered that object, because it would have reminded me of my grandchild's drawing yesterday”. This type of inference appears less likely in people with memory problems (or else more likely in motivated, super-normal elders).

Finally, in the Object-Scene paradigm, the individuals with hippocampal lesions (HL group) tended to show deficits in both Source and Item memory relative to the healthy Older group (consistent with Shimamura & Squire, 1987). However, this was clearly not true for all three patients, with HL3 showing numerical estimates of Source and Item memory that were actually above the control mean. There was no obvious difference about this patient (who is equally amnesic under conventional neuropsychological tests (Henson, Greve, et al., 2016), and of an age, 62, not far from the Older group, 63–82), except that he did not quite finish the second run of the Object-Scene paradigm. Though this was only a loss of 10% of trials overall, these were the trials most susceptible to any build-up of interference or general fatigue. Then again, patient HL2 only completed the first run, yet still exhibited worse memory. If the hippocampal lesions were truly selective (as claimed in Henson, Greve, et al., 2016), then this pattern of reduction in the *Di* as well as *Ds* parameter of the Source-Item model is inconsistent with the anatomical model sketched above, which would predict no reduction in the *Di* parameter if perirhinal cortex is intact (and retrieval from hippocampus and perirhinal cortex is exclusive). It is therefore important to replicate these MPT results in larger numbers of patients, ideally including the converse case of patients with selective lesions of perirhinal cortex (e.g., Bowles et al., 2007).

### 4.4 Caveats

There are two final caveats associated with our paradigms. Firstly, the Object-Scene paradigm is not a classic source memory task, in that the scene (which is seen twice) can be used to cue recall the original location (source), even if the object (item) is forgotten (i.e., it is more like a test of associative memory). For example, a participant may remember something was on the left of the hotel corridor scene, even if they cannot remember what it was. This could be tested by further MPTs that allow for correct source decisions without memory for the item, perhaps by distinguishing two *Ds* parameters, one corresponding to the object cue and one to the scene cue. This leads to the second caveat, namely that our Object-Location paradigm, which is a more classic source monitoring task, cannot measure these cases where Source memory is correct but Item memory is not. Indeed, Tree and Perfect (2004) develop such a paradigm, in which factual details (Item memory) were probed along with their source (seen, read or heard). Accuracy measures of both Source memory conditional on Item memory, and Item memory conditional on Source memory, were impaired with ageing, supporting the associative hypothesis of Naveh-Benjamin (2000), rather than an age-related problem selective to source information per se. This raises the more general point that increasing the range of paradigms (specifically the range of response categories) is necessary to make progress in distinguishing a larger range of possible models of Item and Source memory.

### 4.5 Conclusion

The inferences one makes from source monitoring paradigms depend on the scoring methods used. It is therefore crucial to consider the assumptions underlying a specific scoring method. MPT models are generally preferable to standard Pr calculations, assuming there are sufficient data to fit them. But even with MPT models, at least two different parameterisations of Item and Source memory are possible. These cannot always be distinguished formally in terms of overall model fit, but the consistency of their conclusions (e.g., in terms of significant differences between groups) with other evidence (e.g., from brain data) can be used to favour one MPT model over others.
