# Supplementary material for: Assumptions behind scoring source versus item memory: Effects of age, hippocampal lesions and mild memory problems
Source: Cortex. 2017 Jun;91:297–315. doi: 10.1016/j.cortex.2017.01.001 (PMC5460522; doi:10.1016/j.cortex.2017.01.001)
Supplement: Supplementary file 1 [file mmc1.pdf]

# Supplementary Material

## *Supplementary Proof*

### Paradigm 1

Here we show the mathematical equivalence (isomorphism) between the Item-Source and Source-Item models for Paradigm 1, in terms of how the $D_s$ and $D_i$ parameters relate across the two models.

The MPTs for "New Items" are identical in Item-Source and Source-Item models (Figure 1D). Because $D_n$ cannot be estimated uniquely, it was set to 0. Then $G_i$ is uniquely determined by the *N-N* response category (correct rejections), and so $G_s$ is also uniquely determined (provided $G_i \neq 0$) by the false alarm categories (*N-S1* and *N-S2*), and therefore the estimates of $G_i$ and $G_s$ are identical across models.

Now if $D_s^A$, $D_i^A$ represent parameters from the Item-Source MPT in Figure 1A, and $D_s^B$, $D_i^B$ represent parameters from the Source-Item MPT in Figure 1B, then by equating the "Miss" responses:

$$(1-D_i^A)\times(1-G_i) = (1-D_s^B)\times(1-D_i^B)\times(1-G_i)$$

and thus cancelling $(1-G_i)$:

$$D_i^A = 1-(1-D_s^B)\times(1-D_i^B) = D_s^B + D_i^B - D_s^B D_i^B \qquad [1]$$

By equating the "Src Inc" responses in Figures 1A and 1B:

$$D_i^A \times (1-D_s^A)\times(1-G_s) + (1-D_i^A)\times G_i \times(1-G_s) = \ldots$$
$$(1-D_s^B)\times D_i^B \times(1-G_s) + (1-D_s^B)\times(1-D_i^B)\times G_i \times(1-G_s)$$

and thus cancelling $(1-G_s)$:

$$D_i^A \times(1-D_s^A) + (1-D_i^A)\times G_i = (1-D_s^B)\times D_i^B + (1-D_s^B)\times(1-D_i^B)\times G_i$$

Then expanding:

$$D_i^A - D_i^A D_s^A + G_i - G_i D_i^A = D_i^B - D_i^B D_s^B + G_i - G_i D_s^B - G_i D_i^B + G_i D_s^B D_i^B$$

cancelling $G_i$ term, and gathering $D_i^A$ terms on left:

$$D_i^A(1-D_s^A - G_i) = D_i^B - D_i^B D_s^B - G_i\,(D_s^B + D_i^B - D_s^B D_i^B)$$

Then substituting Equation [1]:

$$(1-D_s^A-G_i)(D_s^B+D_i^B-D_s^B D_i^B)=D_i^B-D_i^B D_s^B-G_i\,(D_s^B+D_i^B-D_s^B D_i^B)$$

One sees that the term $G_i\,(D_s^B+D_i^B-D_s^B D_i^B)$ cancels from both sides, leaving:

$$(1-D_s^A)(D_s^B+D_i^B-D_s^B D_i^B)=D_i^B-D_s^B D_i^B$$

Thus

$$D_i^B+D_s^B-D_i^B D_s^B-D_s^A(D_i^B+D_s^B-D_i^B D_s^B)=D_i^B-D_i^B D_s^B$$

and the terms $D_i^B$ and $D_s^B D_i^B$ cancel from both sides, leaving

$$D_s^B-D_s^A(D_i^B+D_s^B-D_i^B D_s^B)=0$$

In other words:

$$D_s^A=D_s^B\,/\,(D_i^B+D_s^B-D_i^B D_s^B)=D_s^B\,/\,D_i^A \qquad [2]$$

## Paradigm 2

The MPTs for "New Items" for Paradigm 2 are also identical in Item-Source and Source-Item models (Figure 2B and 3B), and the parameter $D_n$ is uniquely determined by the *N-N, High Confidence correct rejection* response category, and therefore estimated identically across models (as apparent in Figure 9). However, unlike in Paradigm 1, the parameters $G_i, G_s$ now also depend on the $D_f$ parameter, so are no longer uniquely determined by a response category, and their estimates can therefore differ across model fits. The only other parameter uniquely determined by a response category is $D_m$ (determined by Misses), and its estimate was identical in all participants except the rare participant for whom $G_i=1$, when $D_m$ can take any value (which is why the pattern of mean values for $D_m$ was not quite identical across models in Figure 9).

## *Supplementary tables*

### Supplementary Table1: Mean, Minimum and Maximum counts across participants for each condition in the Object-Scene experiment

| Condition (trial type) | Response | Young | | Older | | HL1 | HL2 | HL3 |
|---|---|---|---|---|---|---|---|---|
| | | MEAN | [Min, Max] | MEAN | [Min, Max] | Count | Count | Count |
| 2nd Stay | 2nd Stay | **101.17** | [56, 121] | **96.94** | [77, 119] | 72 | 39 | 87 |
| | 2nd Move | **14.83** | [0, 53] | **16.61** | [2, 38] | 30 | 5 | 18 |
| | First | **7.00** | [0, 20] | **8.72** | [1, 23] | 20 | 18 | 5 |
| 2nd Move | 2nd Stay | **42.83** | [15, 67] | **59.28** | [38, 85] | 57 | 30 | 44 |
| | 2nd Move | **69.67** | [43, 107] | **50.44** | [19, 73] | 28 | 7 | 55 |
| | First | **10.50** | [1, 28] | **12.50** | [1, 28] | 39 | 24 | 8 |
| First | 2nd Stay | **15.06** | [1, 51] | **29.78** | [4, 90] | 22 | 7 | 24 |
| | 2nd Move | **10.56** | [0, 80] | **11.72** | [3, 90] | 10 | 0 | 13 |
| | First | **220.39** | [134, 244] | **203.67** | [143, 238] | 214 | 116 | 189 |
| | Maximum possible | 492 | | 492* | | 492 | 246 | 443 |

Supplementary Table1. Mean, Minimum and Maximum counts across participants for each condition in the Object-Scene experiment. The mean count along with the minimum and maximum counts in brackets [Min, Max] is provided for the young and older groups per condition. Individual counts are provided for the individuals with hippocampal lesion individuals (HL1-HL3). * the maximum count for one older participant was 453, owing to a shorter second block.

## Supplementary Table2: Mean, Minimum and Maximum counts across participants for each condition in the Object-Location experiment

| Condition (trial type) | Response | Young | | Older | | MMP | |
|---|---|---|---|---|---|---|---|
| | | MEAN | [Min, Max] | MEAN | [Min, Max] | MEAN | [Min, Max] |
| Bottom | Low Confidence Bottom | **7.33** | [1, 16] | **5.83** | [0, 16] | **12.04** | [0, 31] |
| | High Confidence Bottom | **23.67** | [11, 35] | **21.50** | [6,34] | **9.19** | [0, 31] |
| | Low Confidence Top | **5.33** | [1, 12] | **4.17** | [0,11] | **10.68** | [0, 37] |
| | High Confidence Top | **2.17** | [0, 8] | **5.33** | [0,16] | **4.39** | [0, 27] |
| | Low Confidence New | **0.75** | [0, 3] | **1.00** | [0, 4] | **1.85** | [0, 13] |
| | High Confidence New | **0.33** | [0, 2] | **1.67** | [0, 5] | **0.95** | [0, 7] |
| Top | Low Confidence Bottom | **2.42** | [0, 7] | **3.42** | [0, 12] | **7.80** | [0, 19] |
| | High Confidence Bottom | **1.67** | [0, 6] | **4.58** | [0, 15] | **3.05** | [0, 23] |
| | Low Confidence Top | **10.67** | [2, 18] | **6.25** | [0, 15] | **14.75** | [0, 36] |
| | High Confidence Top | **23.58** | [9, 31] | **23.92** | [12, 35] | **10.63** | [0, 32] |
| | Low Confidence New | **0.67** | [0, 4] | **0.42** | [0, 2] | **1.64** | [0, 12] |
| | High Confidence New | **1.00** | [0, 2] | **1.00** | [0, 4] | **1.30** | [0, 13] |
| New | Low Confidence Bottom | **0.42** | [0, 2] | **0.33** | [0, 2] | **0.49** | [0, 3] |
| | High Confidence Bottom | **0.17** | [0, 1] | **0.92** | [0, 4] | **0.11** | [0, 3] |
| | Low Confidence Top | **0.25** | [0, 2] | **0.25** | [0, 1] | **0.93** | [0, 6] |
| | High Confidence Top | **0.17** | [0, 1] | **0.08** | [0, 1] | **0.16** | [0, 2] |
| | Low Confidence New | **5.92** | [0, 23] | **3.75** | [0, 16] | **11.64** | [0, 40] |
| | High Confidence New | **33.00** | [15, 40] | **34.67** | [24, 40] | **26.25** | [0, 40] |
| | Maximum Possible | 120 | | 120 | | 120 | |

Supplementary Table 2. Mean, Minimum and Maximum counts across participants for each condition in the Object-Location experiment. The mean count along with the minimum and maximum counts in brackets [Min, Max] is provided for the Young, Older and Older with Mild Memory Problems (MMP) groups.
